# From pathways to prediction: a comparative machine learning framework for prostate cancer survival

**DOI:** 10.1093/bioadv/vbag093

**Published:** 2026-03-28

**Authors:** Elif Kardelen Çağdaş, Hüseyin Şan, Berkay Çağdaş, Emre Hafızoğlu

**Affiliations:** Institute of Biotechnology, Ankara University, Ankara, 06135, Türkiy; Department of Pathology, Afyonkarahisar State Hospital, Afyonkarahisar, 03030, Türkiye; Department of Nuclear Medicine, Ankara Bilkent City Hospital, Ankara, 06800, Türkiye; Department of Nuclear Medicine, Afyonkarahisar State Hospital, Afyonkarahisar, 03030, Türkiye; Institute of Nuclear Sciences, Ege University, İzmir, 35100, Türkiye; Department of Oncology, Afyonkarahisar State Hospital, Afyonkarahisar, 03030, Türkiye

## Abstract

**Motivation:**

Prostate cancer shows substantial clinical and molecular heterogeneity, limiting the prognostic accuracy of conventional clinicopathologic models. Single-gene alterations and tumor mutational burden provide limited prognostic discrimination. Pathway-level genomic abstraction may better capture cumulative oncogenic disruption.

**Results:**

Genomic and clinical data from 2231 prostate adenocarcinoma patients were analyzed by mapping somatic mutations to 11 cancer-related signaling pathways. A composite pathway-based risk score integrating pathway burden, p53 pathway status, and high-risk co-alterations was developed and evaluated using survival analysis, Cox regression, time-dependent receiver operating characteristic curves, and machine-learning models, with generalizability assessed in an independent external cohort. The score stratified patients into distinct risk groups with significantly different overall survival (log-rank P < .0001); each one-point increase was associated with a 31% higher mortality risk (hazard ratio 1.31, 95% confidence interval 1.21–1.42). The model showed moderate discrimination (concordance index 0.5897) and more stable predictive performance than tumor mutational burden alone. Machine-learning models achieved similar performance, and feature importance analysis identified p53 pathway disruption and pathway burden as key predictors. The proposed framework is a mutation-based genomic risk-stratification tool derived from targeted-sequencing data that provides interpretable prognostic stratification with performance comparable to machine-learning models.

**Availability and implementation:**

Available upon request.

## 1 Introduction

Prostate cancer represents one of the most frequently diagnosed malignancies among men worldwide and is characterized by marked clinical and molecular heterogeneity (Giona 2021). The disease spans a broad biological spectrum, ranging from indolent, organ-confined tumors to highly aggressive, treatment-resistant variants with widespread metastasis (Culig 2014, Merkens *et al.* 2022). This heterogeneity is driven by a complex interplay of genetic, epigenetic, and microenvironmental factors, culminating in distinct tumor behaviors and therapeutic responses (Wang *et al.* 2018).

Conventional risk-stratification tools—such as PSA levels, Gleason grading, clinical stage, and imaging—remain central to disease management. However, these tools primarily reflect morphological or anatomical features and may not fully capture the underlying molecular complexity of individual tumors (Fernandez-Mateos *et al.* 2024). As a result, patients with seemingly similar clinicopathologic profiles can experience markedly divergent clinical trajectories. This discrepancy underscores the need for integrative molecular strategies capable of improving prognostication and personalizing therapeutic approaches (Ye and Sowalsky 2018).

Recent years have witnessed significant efforts toward molecular subtyping of prostate cancer using transcriptional classifiers (e.g. PAM50), genomic aberration patterns, and integrative multi-omic data (Yoon *et al.* 2021, McCabe *et al.* 2024). Notably, studies have identified recurrent alterations in genes such as TP53, PTEN, SPOP, FOXA1, and RB1, as well as structural rearrangements involving ERG and other ETS family members (Cancer Genome Atlas Research Network 2015, Cotter and Rubin 2022). Despite these advances, many of these molecular signatures are yet to be translated into routine clinical risk models due to their complexity, limited reproducibility, and lack of robust clinical validation.

To overcome the sparsity and inter-patient heterogeneity of gene-level mutational data, pathway-based approaches have emerged as a promising alternative. These methods group mutations into predefined pathways, enabling functional abstraction and meaningful biological stratification (Chen *et al.* 2024). Prior studies have shown that cumulative pathway disruption—particularly involving PI3K, TP53, RTK/RAS, and DNA repair networks—correlates with disease aggressiveness and poor survival in multiple cancers, including prostate (McCabe *et al.* 2024).

Furthermore, key genomic alterations such as TP53 inactivation have consistently been linked to poor prognosis, therapy resistance, and castration-resistant disease evolution (Ofner *et al.* 2025). Similarly, co-alterations in PIK3CA and MAPK signaling components have been implicated in treatment failure and lineage plasticity (He *et al.* 2022). These observations suggest that integrative scoring systems, which consider the cumulative and combinatorial effects of pathway-level genomic changes, may provide enhanced prognostic power and better reflect underlying tumor biology.

Although several genomic classifiers and molecular stratification systems have been proposed in prostate cancer, most established tools are transcriptome-based, proprietary, or optimized for localized disease settings, including Decipher, Prolaris, Oncotype DX GPS, PAM50, and PCS. These platforms have demonstrated prognostic utility, but they are not primarily designed as mutation-only, pathway-constrained, interpretable models that can be directly reconstructed from routine targeted DNA sequencing panels. Likewise, aggressive-variant frameworks centered on TP53, RB1, and PTEN capture highly adverse biology, but typically focus on selected tumor-suppressor events rather than integrating pathway burden and co-alteration architecture into a compact additive score. PIRSP-PC was therefore conceived not as a replacement for existing genomic classifiers, but as a complementary, mutation-based and biologically transparent framework intended to bridge functional pathway abstraction with practical deployability (Zhao *et al.* 2017, Jairath *et al.* 2021, Yoon *et al.* 2021, Pedrani *et al.* 2025).

### 1.1 Conceptual benchmarking against existing genomic stratification frameworks

Several genomic risk-stratification frameworks have been developed to improve prognostic assessment in prostate cancer beyond conventional clinicopathologic parameters. These tools generally fall into two broad methodological categories: transcriptome-based genomic classifiers and mutation-driven molecular frameworks. Expression-based classifiers—such as Decipher, Prolaris, and the Oncotype DX Genomic Prostate Score—derive prognostic information from tumor RNA expression profiles and have demonstrated clinical utility in refining risk assessment and informing treatment decisions in localized prostate cancer settings. These assays typically quantify transcriptional programs associated with proliferation, androgen signaling, stromal response, and tumor aggressiveness, and generate continuous risk scores intended to estimate the likelihood of adverse pathology, biochemical recurrence, or metastatic progression (Loeb and Ross 2017).

Other molecular frameworks focus on subtype classification or tumor-suppressor gene architecture rather than continuous genomic scoring. Transcriptomic subtype systems such as PAM50 or PCS attempt to capture biologically distinct transcriptional states within prostate tumors, enabling molecular taxonomy of luminal and basal phenotypes and their associated clinical behavior. In contrast, mutation-oriented frameworks—including aggressive-variant prostate cancer (AVPC) models—emphasize combined alterations in key tumor-suppressor genes such as TP53, RB1, and PTEN, which together define a subset of biologically aggressive disease characterized by therapy resistance and poor clinical outcomes (Garcia de Herreros *et al.* 2024).

Despite their demonstrated prognostic relevance, these approaches differ substantially in data modality, analytical design, and translational applicability. Expression-based genomic classifiers typically require transcriptomic profiling and platform-specific normalization procedures, which may limit portability across sequencing technologies and clinical environments. Conversely, mutation-centered frameworks are more readily compatible with routine targeted-sequencing panels but often focus on a limited number of genes or tumor-suppressor alterations rather than capturing broader pathway-level genomic architecture.

To contextualize the present study within this methodological landscape, we performed a conceptual benchmarking analysis comparing PIRSP-PC with representative genomic classifiers and pathway-oriented models reported in the prostate cancer literature. Rather than attempting direct numerical performance comparisons—which are not feasible across fundamentally different data modalities—we focused on key conceptual dimensions including data modality, biological abstraction level, interpretability, sequencing compatibility, and clinical deployment context. The resulting comparison is summarized in Table 1, highlighting how PIRSP-PC differs from existing frameworks in its mutation-based design, pathway-constrained architecture, and rule-based interpretability.

**Table 1:** Conceptual comparison of PIRSP-PC with representative genomic and pathway-based risk-stratification frameworks in prostate cancer.

Framework	Data type	Core biological basis	Output	Interpretability	Requires transcriptomics	Applicable to targeted DNA panels	Primary intended setting	Main limitation	Distinguishing feature relative to PIRSP-PC
Decipher	RNA expression (22-gene signature)	Gene expression program associated with metastatic potential and tumor aggressiveness	Continuous genomic risk score	Moderate (algorithmic model)	Yes	No	Localized prostate cancer after prostatectomy	Proprietary platform; requires expression profiling	Strong clinical validation but not mutation-based
Prolaris	RNA expression (cell-cycle gene panel)	Cell-cycle progression genes reflecting proliferative activity	Continuous risk score	Moderate	Yes	No	Localized disease; treatment decision support	Platform-specific testing required	Focus on proliferation rather than pathway architecture
Oncotype DX GPS	RNA expression (17-gene panel)	Androgen signaling, proliferation, stromal and cellularorganization genes	Genomic Prostate Score (0–100)	Moderate	Yes	No	Localized low- and intermediate-risk prostate cancer	Expression-dependent and proprietary	Designed for biopsy specimens rather than sequencing datasets
PAM50	RNA expression (50-gene classifier)	Molecular subtypes (luminal vs basal) reflecting transcriptional programs	Molecular subtype classification	Moderate	Yes	No	Primarily localized disease; subtype discovery	Requires transcriptomic profiling and normalization	Captures tumor subtype biology rather than mutation architecture
PCS (prostate cancer subtypes)	RNA expression	Integrative expression-based subtype classification	Subtype assignment	Moderate	Yes	No	Molecular taxonomy research	Limited clinical deployment	Focuses on subtype stratification rather than additive genomic risk
AVPC-TSG/TP53-RB1-PTEN frameworks	DNA mutation/copy number	Combined loss of tumor suppressors associated with aggressive-variant prostate cancer	Binary aggressivevariant classification	High	No	Yes	Advanced/castration-resistant disease	Limited to specific tumor-suppressor alterations	Captures aggressive biology but does not incorporate pathway burden
PIRSP-PC	DNA somatic mutation data	Pathway alteration burden, p53 pathway status, and co-alterations involving PI3K or RTK–RAS signaling	Rule-based additive genomic risk score	High	No	Yes	Mixed clinical states in sequencing cohorts	Moderate discrimination; external survival validation limited	Mutation-only, pathway-constrained framework compatible with routine targeted-sequencing panels

In this study, we develop and validate a pathway-centric genomic scoring system using data from the MSK-IMPACT cohort of prostate acinar adenocarcinoma patients. The scoring model integrates three critical dimensions of tumor genomics: the burden of pathway-level alterations, p53 pathway status, and the co-occurrence of high-impact alterations across PI3K, p53, and RTK/RAS axes. We hypothesize that this composite score, hereafter referred to as PIRSP-PC (Pathway Integrative Risk Stratification for Prostate Cancer), will outperform traditional mutation burden metrics.

## 2 Methods

### 2.1 Patient cohort and data acquisition

We retrospectively analyzed genomic and clinical data of 2231 cases from the Memorial Sloan Kettering-Integrated Mutation Profiling of Actionable Cancer Targets that have a variant allele frequency of ≥0.05 [MSK-IMPACT (Cheng *et al.* 2015)] prostate adenocarcinoma cohort, publicly available through cBioPortal (Cerami *et al.* 2012, Gao *et al.* 2013). Patient-level data included demographic variables, clinical parameters (age, tumor stage, Gleason score), molecular features [tumor mutation burden (TMB), microsatellite instability (MSI) status], and survival outcomes. Overall survival (OS) was calculated in months from diagnosis to death or last follow-up. Only patients with complete somatic mutation profiles and survival data were included. Duplicate patient identifiers were screened and removed to avoid potential redundancy within the dataset.

In addition, an independent external validation cohort was obtained from cBioPortal on 6 March 2026, using the Prostate Adenocarcinoma [TCGA, GDC, 2025 (Cancer Genome Atlas Research Network 2015)] dataset, which includes 501 prostate cancer cases. This cohort was used for external genomic validation of the PIRSP-PC framework. Due to the limited number of survival events in the TCGA dataset, formal survival modeling was not performed. The same preprocessing strategy, pathway annotation schema, and score construction rules were applied to the external cohort to evaluate model portability and generalizability.

Patients with missing covariate data required for Cox regression were excluded from survival modeling analyses.

This study utilized fully anonymized, publicly available data from the cBioPortal for Cancer Genomics. As such, no institutional review board approval or informed consent was necessary in accordance with local and international data-sharing regulations. Access to the cBioPortal platform was last performed on 10 June 2025, at 10:00 a.m. and the external validation cohort was additionally retrieved on 6 March 2026.

### 2.2 Pathway annotation and feature matrix construction

We applied a pathway-centric annotation framework to convert gene-level somatic mutation data into functional events. Using the 2024 release of the TCGA Database, each mutated gene was mapped to its corresponding curated signaling or biological pathways. Specifically, we focused on 11 key cancer-related pathways: p53 signaling, PI3K-AKT, RTK–RAS, WNT, cell cycle, DNA damage repair, MYC signaling, Notch, TGF-β, Hippo, and Apoptosis. For each patient, a binary matrix was constructed in which a pathway was marked “1” if it contained at least one nonsynonymous mutation in any member gene, and “0” otherwise. This transformation yielded a sparse binary matrix of dimensions *n* × *p*, where *n* denotes the number of patients and *p* the number of unique pathways.

### 2.3 Construction of the PIRSP-PC

We developed a composite risk scoring system based on three core genomic dimensions:

1. Pathway burden score
2. Patients with 0, 1, or 2 mutated pathways received 0, +1, or +2 points, respectively.
3. Patients with ≥3 mutated pathways received +1 point, recognizing the plateauing effect of extensive alterations.
4. p53 pathway status
5. Presence of any mutation in the p53 signaling pathway (e.g. TP53 inactivation) was assigned +2 points due to its known strong prognostic impact.
6. High-impact co-alteration penalty
7. Patients with co-occurring alterations in p53 and either PI3K or RTK–RAS pathway were assigned an additional +1 point.

The total score (range: 0–5) was used to stratify patients into three risk groups:

- Low risk: score = 0
- Intermediate risk: score = 1
- High risk: score ≥ 2

This scoring system aims to achieve a balance between biological interpretability and predictive precision.

The PIRSP-PC was constructed using biologically grounded logic in conjunction with empirical performance metrics derived from univariable survival analyses. Each component was weighted according to its observed prognostic relevance and mechanistic plausibility:

Pathway burden: The identification of mutations in molecular pathways is contingent upon the detection of any genetic alterations within the pertinent pathway. The tumor mutation load was determined by the total count of nonsynonymous mutations identified in the tumor tissue. The count of affected pathways denotes the total pathways in which mutations were identified in a patient. Patients were assigned 0 points if no pathway was altered, +1 for a single-pathway alteration, and +2 if two pathways were altered.p53 pathway alteration (+2 points): The p53 signaling axis has consistently demonstrated strong association with adverse outcomes in prostate cancer. In our dataset, p53 pathway alterations conferred a significantly higher hazard ratio (HR) of 1.65 for OS, and yielded an AUC of 0.618 at 12 months, outperforming other single-pathway metrics. Based on this consistently elevated risk and its well-established biological role in genomic instability and therapy resistance, it was assigned the highest individual weight.

Co-alteration of p53 with PI3K or RTK–RAS (+1 point): The presence of concurrent alterations in p53 and either PI3K or RTK–RAS signaling components was associated with poorer prognosis (HR: 1.42; *P* < .01), suggesting synergistic biological interaction and additive risk. This co-alteration reflects the convergence of cell-cycle deregulation and mitogenic signaling, and thus warranted additional weighting.

Alternative scoring strategies, including unweighted additive models and machine-learning classifiers (e.g. random forest, LASSO), were preliminarily explored. However, they offered only marginal improvements in AUC (≤0.01 gain) and considerably reduced interpretability. Therefore, this semi-weighted additive scoring scheme was selected to optimize biological relevance, clinical usability, and model transparency.

To determine the prognostic contribution of individual pathways, univariate Cox regression was performed on 11 functionally annotated signaling pathways. This allowed us to empirically identify the most impactful features for inclusion in the PIRSP-PC. Specifically, pathways with statistically significant associations (*P* < .05) and HRs indicating increased mortality were prioritized.

After development in the MSK-IMPACT cohort, the finalized PIRSP-PC scoring system was applied without modification to the external TCGA-GDC cohort for validation.

#### 2.3.1 Machine learning-based survival modeling and SHAP interpretability

To benchmark the prognostic performance of the pathway-based score against more flexible, non-linear modeling approaches, we constructed random survival forest (RSF) and gradient boosting survival analysis (GBSA) models using the same input variables (binary pathway mutation flags, TMB, and Affected_Pathway_Count). For machine-learning models, the dataset was randomly divided into a stratified training and testing subset using a 70/30 split. Model training and hyperparameter optimization were performed on the training set, while predictive performance was evaluated on the held-out test set. Model training and evaluation were conducted and performance was assessed via *C*-index, Brier score, integrated Brier score (IBS), time-dependent AUC, and log.

To enhance interpretability, SHAP (SHapley Additive exPlanations) values were computed for each model, quantifying the relative importance of genomic features in survival prediction. Comparative significance of SHAP rankings was tested using paired *t*-tests.

### 2.4 Time-dependent ROC analysis

To evaluate the prognostic discriminative ability of the PIRSP-PC over time, we conducted time-dependent receiver operating characteristic (ROC) analysis using cumulative/dynamic AUC methodology. AUCs were computed at predefined intervals (12, 24, 36, 48, 60, and 72 months) for the following predictors:

- Total PIRSP-PC.
- Risk group (low, intermediate, high).
- p53 pathway alteration status (binary).
- Pathway burden score (pathways mutated count).
- TMB, computed as the number of distinct mutated genes.

Comparative performance of these predictors was visualized through time-AUC plots.

### 2.5 Survival and statistical analyses

Survival curves were generated using the Kaplan–Meier method and compared via the log-rank test. Univariable and multivariable Cox proportional hazards regression models were used to quantify the association between genomic subtypes, risk scores, and OS. Covariates in multivariable models included age, Gleason score, stage, MSI status, and TMB. Proportional hazards assumptions were assessed using Schoenfeld residuals. The interaction terms (e.g. Risk Group × Stage) were explored but excluded due to non-significant improvement in model fit.

Pathway-level enrichment analysis was conducted via chi-square testing across molecular subtypes with Benjamini–Hochberg correction (FDR < 0.05). Fisher’s exact test was employed to assess mutual exclusivity and co-occurrence patterns among frequently altered genes. Mutation sparsity and frequency were visualized using oncoprints and heatmaps.

We employed multiple statistical approaches, including methods beyond conventional ROC analysis, to evaluate the discriminative ability of the proposed risk model. In addition to time-dependent AUC, we calculated the Harrell’s *C*-index to assess concordance between predicted risk and observed survival ranking. Furthermore, net reclassification improvement (NRI) was computed to quantify the gain in classification accuracy when transitioning from simpler models (e.g. p53 status or TMB alone) to the PIRSP-PC. Kaplan–Meier analyses included log-rank tests, median OS estimates, and HRs derived from both univariable and multivariable Cox regression models. To mitigate overfitting and assess model stability, we conducted bootstrap resampling (*n* = 1000 iterations) for the rule-based PIRSP-PC score and implemented a stratified 70/30 train—test split for machine-learning models (RSF and GBSA).

RSF and GBSA models have been employed for survival analysis utilizing machine-learning techniques. The layered cross-validation technique was utilized for hyperparameter adjustment and performance assessment of the models. Metrics including the concordance index (*C*-index), Brier score, IBS, time-dependent AUC, and log-rank test have been employed to assess model performance. Because the primary analytical objective was time-to-event modeling rather than fixed-time binary classification, confusion-matrix—based metrics (e.g. accuracy, precision, recall, or *F*1-score) were not calculated. Furthermore, SHAP analysis has been performed to elucidate the models’ predictions and assess variable significance.

### 2.6 Software and computational environment

All analyses were conducted using Python (v3.11) and R (v4.3), employing the following packages:

- Python: pandas, scikit-learn, lifelines, matplotlib, seaborn, scikit-survival, shap, numpy.
- R: survival, survminer, timeROC, ggplot2.

## 3 Results

### 3.1 MSK-IMPACT data

#### 3.1.1 Patient cohort and clinical characteristics

A total of 2231 prostate adenocarcinoma patients from the MSK-IMPACT dataset were included in the final analysis. The median age at diagnosis was 71 years [interquartile range (IQR): 66–77]. The majority of patients were of non-Hispanic White ethnicity. Gleason scores were distributed as follows: 17.3% had Gleason ≤7, 41.1% had Gleason 8, and 41.6% had Gleason ≥9. MSI status was stable in 92.4% of cases, with only a small fraction demonstrating high MSI.

The median OS for the cohort was 40.8 months (range: 19.3–60.1 months), and 39.5% of patients had died by the time of data cutoff. These clinical features reflect a heterogeneous cohort spanning both localized and advanced disease stages.

#### 3.1.2 TMB and gene-level mutation landscape

TMB, defined as the number of distinct mutated genes per patient, was 3 (range: 0–49). Mutation frequency analysis identified several recurrently altered genes (Table 2).

**Table 2:** Top 10 most frequently mutated genes in the MSK-IMPACT prostate cancer cohort.^a^

Gene	Mutated cases (*n*)	Mutation frequency (%)
*TP53*	701	32.3
*PTEN*	552	25.4
*SPOP*	286	12.6
*FOXA1*	239	11.9
*ARID1A*	169	8.3
*RB1*	139	8.0
*PIK3CA*	133	7.4
*BRCA2*	128	6.9
*ATM*	115	6.7
*CDK12*	104	5.1

a TP53 (32.3%) and PTEN (25.4%) were the most prevalent alterations, consistent with their central roles in tumor-suppressor inactivation and genomic instability. Recurrent mutations in SPOP, FOXA1, and ARID1A further underscore the contribution of chromatin regulation, androgen receptor signaling, and lineage-specific transcriptional programs to prostate cancer biology.

The TP53, PTEN, and RB1 genes—well-established tumor suppressors—showed strong enrichment in high-grade and metastatic cases, consistent with their known association with genomic instability and castration resistance.

#### 3.1.3 Co-occurrence and mutual exclusivity of mutated genes

Pairwise Fisher’s exact tests were performed to assess statistically significant co-alteration and mutual exclusivity among frequently mutated genes. Several noteworthy patterns emerged:

- Significant co-occurrence was observed between TP53 and PIK3CA (*P* < .001), as well as between TP53 and RB1(*P* < .001).
- SPOP and TP53 were found to be mutually exclusive (*P* = .004, FDR-adjusted), aligning with prior observations of divergent molecular lineages in prostate cancer.
- Co-alterations of TP53 and PIK3CA were particularly enriched among patients with high TMB and poor survival, suggesting potential synergistic oncogenic effects.

**Figure 1:**
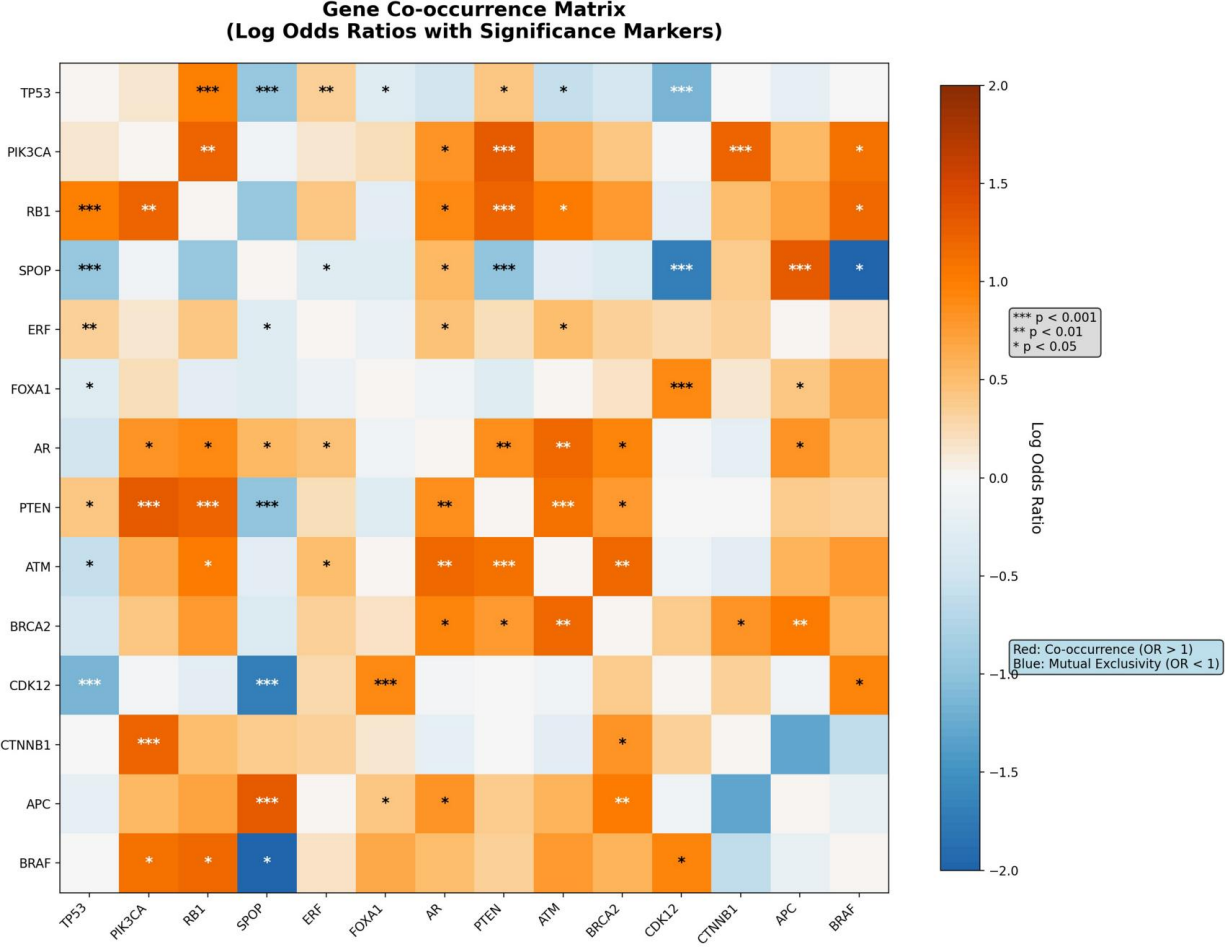
Heatmap displaying pairwise co-occurrence and mutual exclusivity relationships among cancer-related genes in the MSK-IMPACT prostate cancer dataset. The matrix is symmetric, with each cell representing the log odds ratio (OR) for a gene pair. Cells with OR > 1 indicate co-occurrence and cells with OR < 1 indicate mutual exclusivity, with asterisks marking statistically significant pairs (*P < .05, **P < .01, *P < .001).

Gene-level interaction analysis demonstrated distinct co-occurrence and mutual exclusivity patterns among key prostate cancer genes (Fig. 1), highlighting coordinated genomic alterations within high-risk tumors.

These findings support the biological plausibility of co-altered pathway targeting and provided the rationale for incorporating co-alteration status into risk scoring.

#### 3.1.4 Pathway alteration burden across the cohort

Each mutated gene was mapped to one or more TCGA pathways, and pathway-level activation status was recorded per patient (Table 3). The number of altered pathways per patient ranged from 0 to 11, with a median of 2.

**Table 3:** Prevalence of canonical pathway alterations in the MSK-IMPACT prostate cancer cohort.^a^

Pathway	Altered in (%) of patients
p53 signaling	33.4
PI3K pathway	16.6
WNT pathway	13.4
RTK–RAS pathway	10.3

a Alterations in the p53 pathway were most prevalent (33.4%), followed by PI3K (16.6%), WNT (13.4%), and RTK–RAS (10.3%) signaling. These pathways govern fundamental processes including genomic integrity, proliferation, differentiation, and growth-factor signaling. Their high alteration rates highlight their central role in prostate tumorigenesis and support their selection as core elements of the pathway-based risk-stratification framework.

The distribution of altered pathways formed the basis for the burden component of the PIRSP-PC.

#### 3.1.5 Construction and distribution of the PIRSP-PC

The performances of the RSF and GBSA models were compared in the context of machine learning-based survival analysis. The mean *C*-index value of the RSF model was determined to be 0.6082 (SD = 0.0122), whereas the GBSA model had a value of 0.6026 (SD = 0.0141). The Brier score averaged 0.1877 for RSF and 0.1906 for GBSA; the IBS was determined to be 0.1912 for RSF and 0.1939 for GBSA. The time-dependent AUC values averaged 0.6174 for RSF and 0.6119 for GBSA. The log-rank test comparison of the risk groups yielded an average of *P* = .0002 for RSF and *P* = .0010 for GBSA. The paired *t*-test studies for inter-model measures revealed no significant differences in *C*-index (*P* = .4029), Brier score (*P* = .0594), IBS (*P* = .0805), AUC (*P* = .5269), and log-rank *P* (*P* = .3717).

In the assessment of variable impacts on survival by SHAP analysis, the p53 pathway mutation, TMB, and affected pathway count were identified as the most significant variables in both the RSF and GBSA models. In the RSF model, the mean SHAP values were determined to be 32.08 for p53, 15.10 for TMB, and 8.20 for the affected pathway count. In the GBSA model, the values were 0.233, 0.154, and 0.039, respectively. The SHAP values for the remaining pathways were diminished in both models.

**Figure 2:**
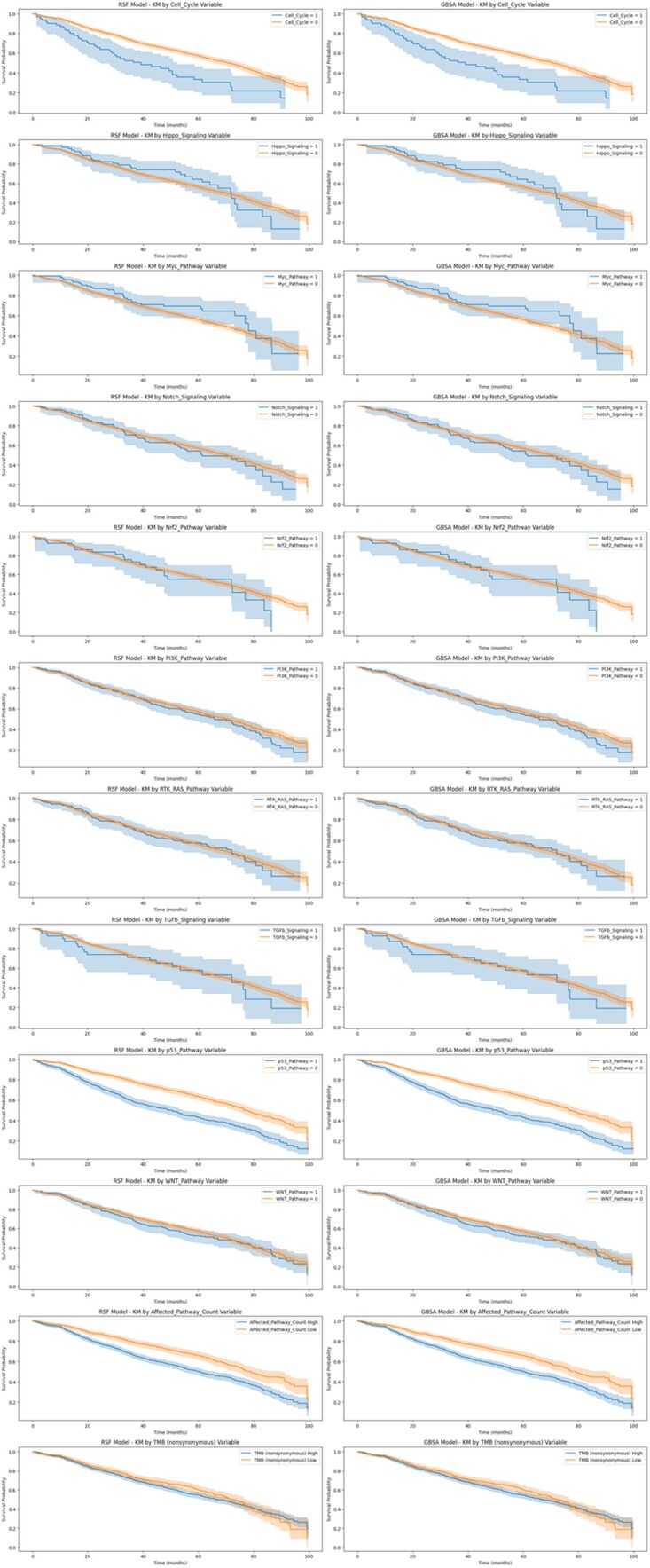
Shapley Additive Explanations summary plot for the random survival forest model, ranking features by contribution to predicted mortality risk, with p53 pathway disruption and pathway burden identified as the strongest predictors.

Upon comparison of the modified SHAP values across the models, it was seen that TMB had a markedly greater contribution in the GBSA model relative to the RSF (*P* = .0205), whereas the number of affected pathways, including the RTK–RAS and WNT pathways, were more pronounced in the RSF model (*P* < .05). The p53 pathway’s impact was comparable in both models (*P* = .1226) (Table 4, Fig. 2).

**Table 4:** Comparison of adjusted SHAP importance scores between RSF and GBSA models.^a^

Feature	RSF adj SHAP (%)	GBSA adj SHAP (%)	*t*	*P*
TMB	22.65	29.50	−3.72	.0205
Affected pathway count	12.67	7.40	4.82	.0085
RTK/RAS pathway	1.27	0.95	2.82	.0478
WNT pathway	1.36	0.73	2.97	.0411
p53 pathway	48.90	44.90	1.95	.1226
Cell cycle	5.85	6.80	−1.77	.1521
PI3K pathway	1.99	2.48	−1.46	.2190
Myc pathway	2.04	2.24	−0.34	.7507
Notch signaling	1.11	1.98	−1.67	.1694
Nrf2 pathway	0.57	1.32	−1.66	.1724
Hippo signaling	1.09	1.22	−0.42	.6985
TGFb signaling	0.49	0.47	0.18	.8695

a SHAP scores represent the relative contribution of each pathway to survival prediction, with positive *t*-values indicating greater importance in RSF. Statistically significant differences (*P* < .05) denote pathway-specific divergence in feature prioritization, reflecting distinct algorithmic interpretations of genomic prognostic signals.

The pathway-based risk scoring system achieved a *C*-index of 0.5897 (continuous score) and 0.5856 (for three-group stratification), indicating moderate discriminative ability. Kaplan–Meier analysis revealed statistically significant differences in survival between:

- Low versus high risk (*P* = 6.33e−18).
- Intermediate versus high risk (*P* = 6.14e−08).
- Marginal difference between low versus intermediate (*P* = .0997).

There was a clear inverse relationship between the risk group and survival duration, as well as a direct relationship with mortality (*χ*^2^  *P* < .001) (Table 5).

**Table 5:** Clinical outcomes by PIRSP-PC groupings.^a^

Risk group	Score range	*n*	Median OS (months)	Death rate (%)
Low risk	0	960	79.0	32.1
Intermediate risk	1	473	78.1	37.7
High risk	≥2	798	51.3	53.8

a Patients were classified as low (score = 0), intermediate (score = 1), or high risk (score ≥ 2). The high-risk group exhibited substantially shorter median overall survival and a higher mortality rate compared with the low-risk group, demonstrating the ability of PIRSP-PC to discriminate clinically distinct subgroups with divergent survival outcomes.

#### 3.1.6 Time-dependent ROC analysis

To assess predictive performance over time, we conducted time-dependent ROC analysis at 12, 24, 36, 48, 60, and 72 months. The PIRSP-PC showed consistently higher area under the curve (AUC) values compared to TMB and pathway burden alone (Table 6).

**Table 6:** Time-dependent AUC values comparing prognostic performance of the PIRSP-PC and individual genomic predictors.^a^

Time (months)	AUC (risk score)	AUC (p53 pathway)	AUC (TMB)	AUC (pathway burden)
12	0.613	0.618	0.581	0.576
36	0.608	0.605	0.564	0.578
72	0.576	0.569	0.505	0.556

a The composite PIRSP-PC demonstrated modest but generally more stable discriminative performance compared with individual genomic predictors, including p53 pathway status, tumor mutational burden, and total pathway burden. Although the p53 pathway alone showed marginally higher AUC at 12 months, PIRSP-PC provided improved longitudinal prognostic stability, supporting the advantage of integrated, biologically informed risk modeling over single-feature approaches.

**Figure 3:**
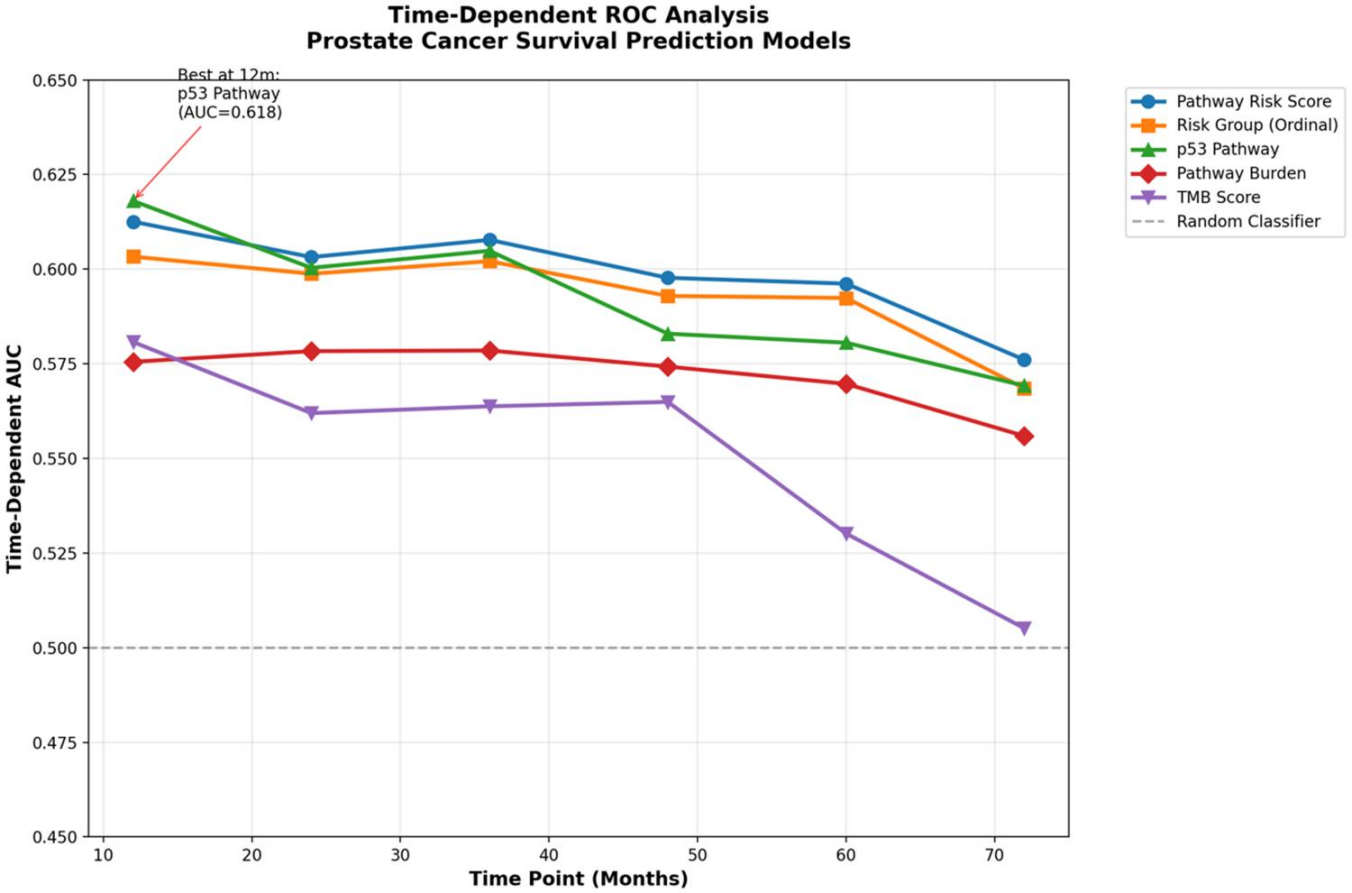
Time-dependent receiver operating characteristic curves comparing five survival prediction models across follow-up time points, illustrating differences in discriminative performance and long-term prognostic stability.

The PIRSP-PC maintained moderate but consistent discriminatory ability across all time points, outperforming traditional genomic metrics (Fig. 3).

### 3.2 Survival outcomes according to risk stratification

Kaplan–Meier survival curves demonstrated significant separation between the risk groups (log-rank *P* < .0001). While the low- and intermediate-risk groups showed similar survival trajectories over the first 36 months, a divergence became more evident over longer follow-up durations. The high-risk group exhibited significantly worse outcomes throughout.

Univariate Cox regression analysis was conducted on 2171 patients, revealing a 41.1% overall mortality rate. Among the 11 tested pathways, three were significantly associated with OS:

- p53 pathway alterations (mean shap: 32.08; HR = 1.311; 95% CI: –; *P* < .0001).
- Affected pathway count (mean shap: 8.20; HR = 1.147; *P* < .0001).
- Cell-cycle pathway alterations (mean shap: 3.91; HR = 1.120; *P* < 0.0001).

These results support the inclusion and weighting of these variables in the final risk score model and reinforce their biological and clinical relevance in prostate cancer prognosis.

**Figure 4:**
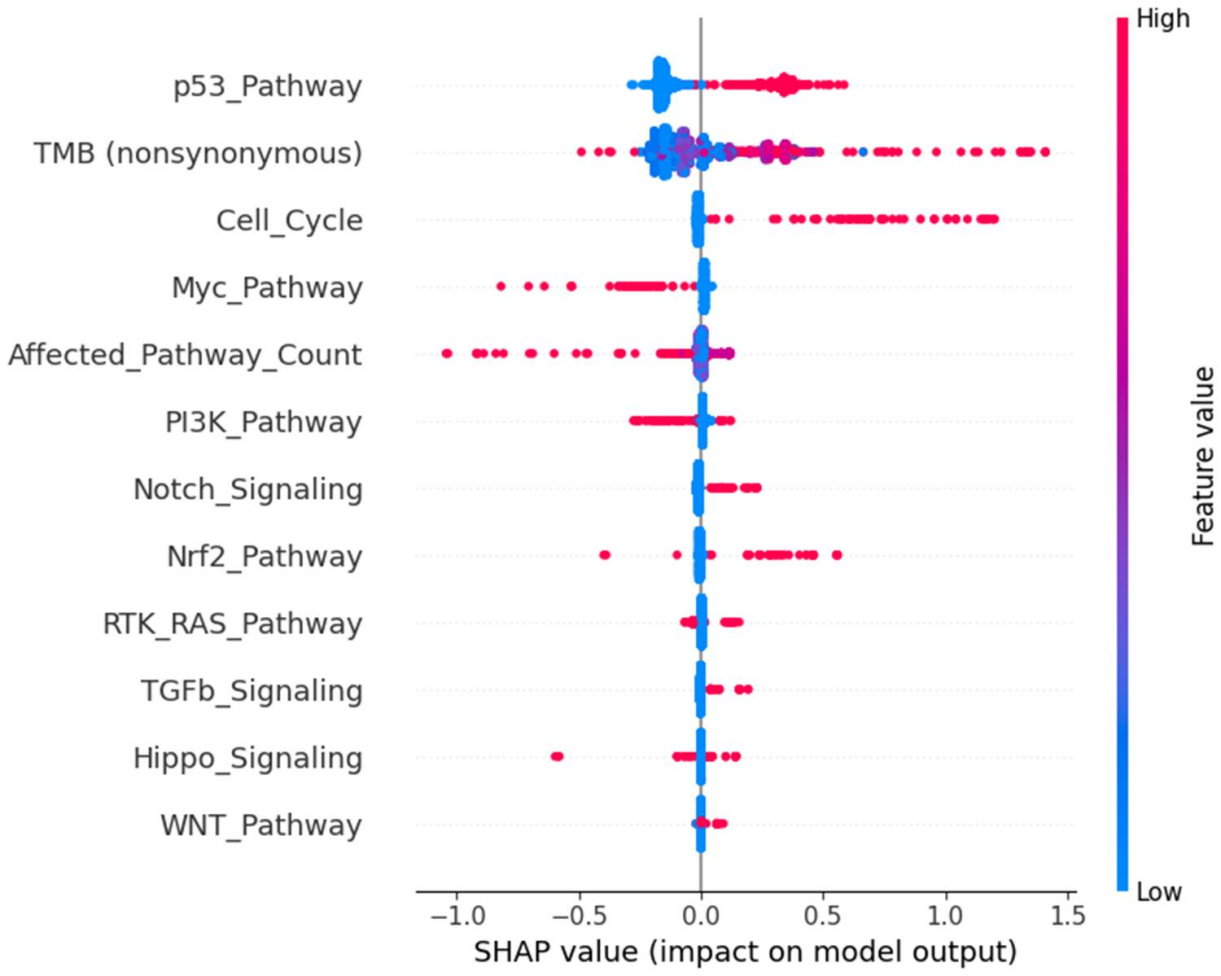
Kaplan-Meier survival curves stratified by alteration status for multiple signalling pathways, shown separately for random survival forest and gradient boosting survival analysis models, with 95 percent confidence intervals over 100 months of follow-up.

Multivariable Cox regression analysis confirmed the independent prognostic value of the PIRSP-PC (HR per point increase: 1.31; 95% CI: 1.21–1.42; *P* < .0001), even after adjusting for age, tumor stage, Gleason score, and TMB (Fig. 4).

To further evaluate the robustness of the categorical risk stratification, a sensitivity analysis was performed using an ordinal representation of the PIRSP-PC risk groups (low = 0, intermediate = 1, high = 2). Kaplan–Meier analysis confirmed significant survival differences across these ordered risk categories (log-rank *χ*^2^ = 6.85, *P* = .0325).

In an ordinal Cox proportional hazards model, each one-step increase in the risk group was associated with a 13% increase in mortality risk (HR = 1.13, *P* = .0136).

Although the discriminative ability of the risk group variable alone was modest (*C*-index = 0.52), the direction and statistical significance of the association support the internal consistency of the PIRSP-PC stratification scheme and indicate that increasing genomic risk categories correspond to progressively worse survival outcomes.

### 3.3 Machine learning model performance

**Figure 5:**
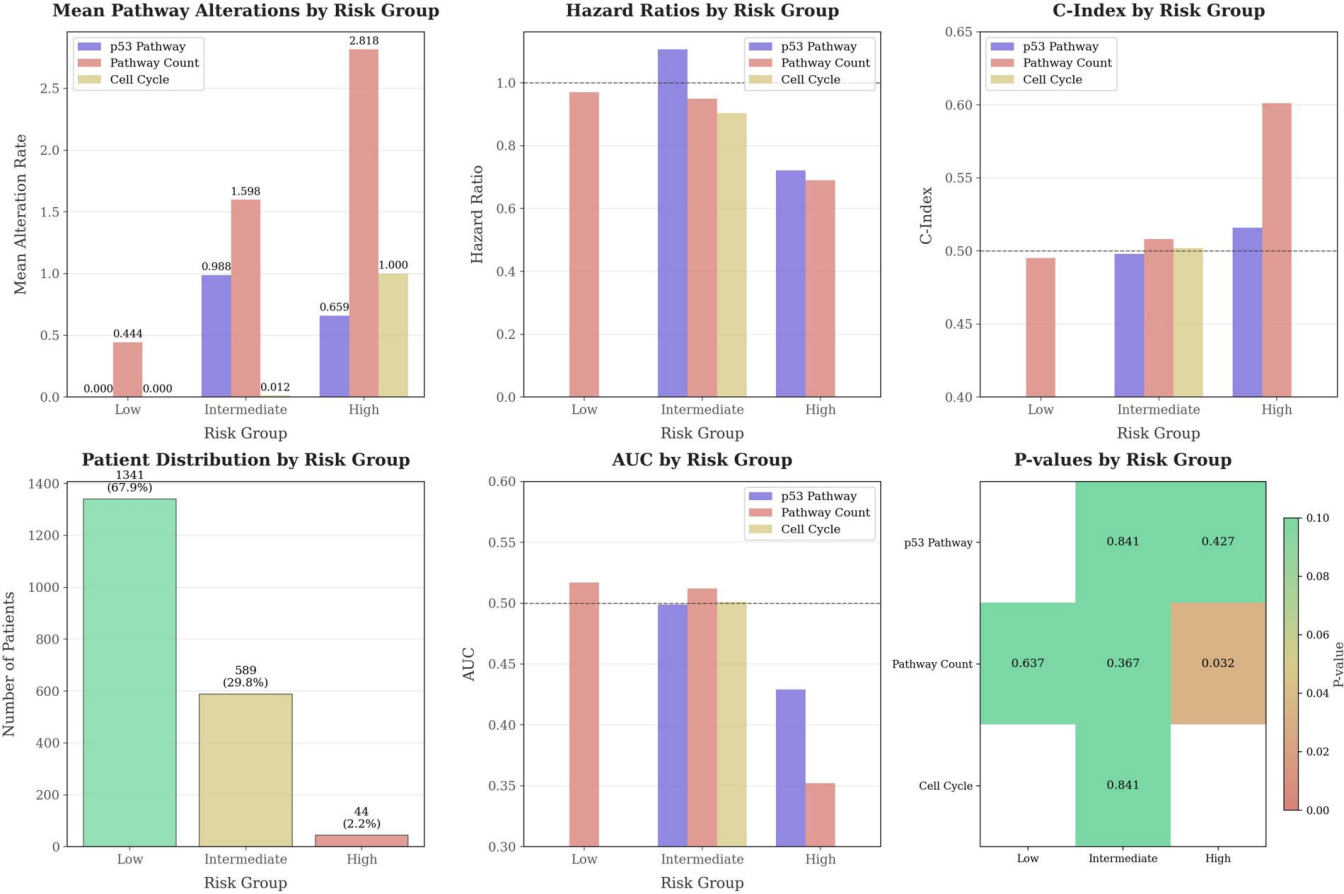
Summary panel displaying pathway alteration rates, hazard ratios, concordance index, and area under the curve values across low-, intermediate-, and high-risk groups, alongside patient distribution by risk stratum.

**Figure 6:**
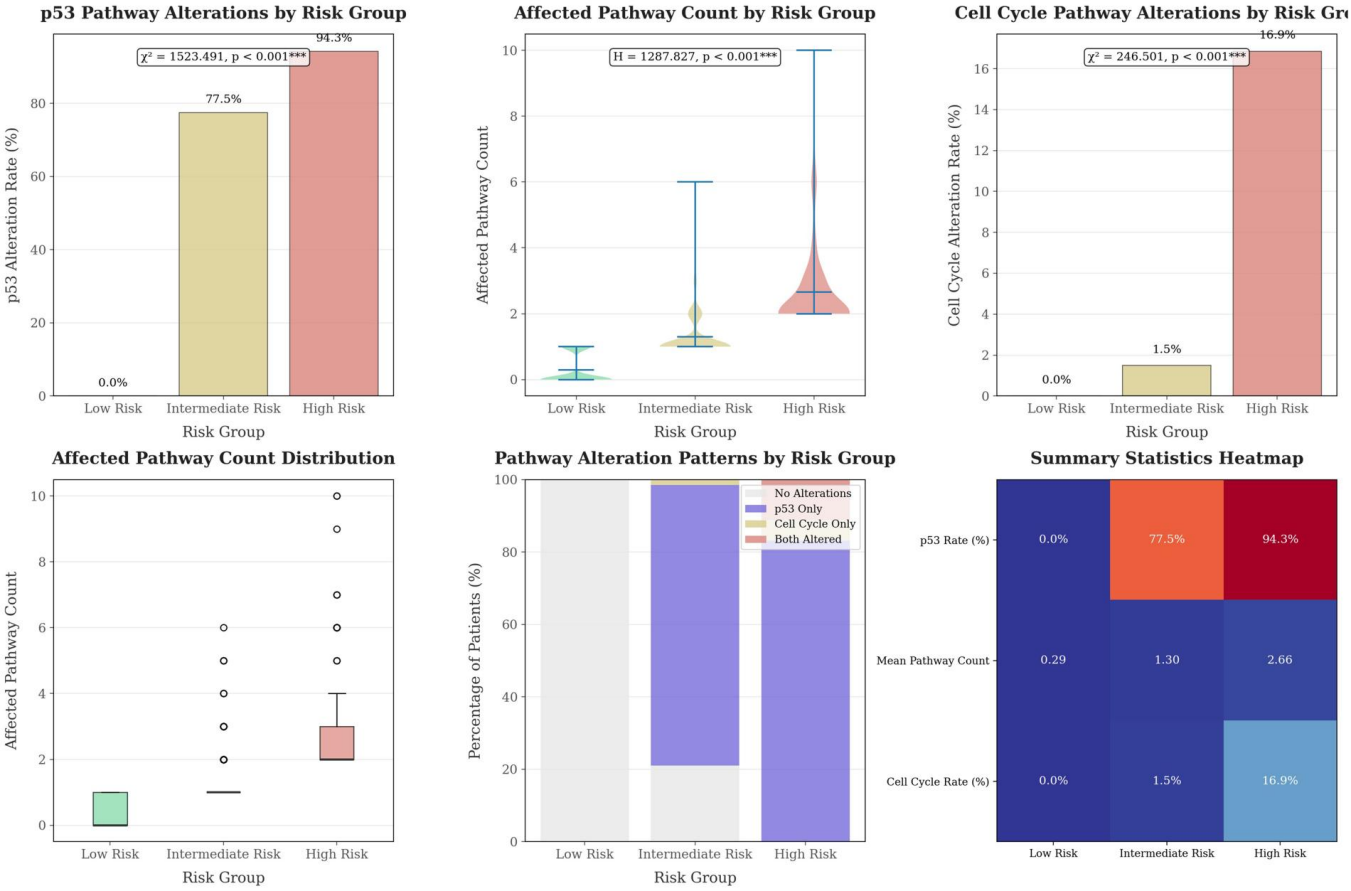
Violin plots, box plots, stacked bar charts, and a summary heatmap showing distributions and patterns of pathway alterations across low-, intermediate-, and high-risk groups, with statistical significance indicated.

Using a 70/30 train—test split, the RSF and GBSA models were trained on the training subset and evaluated on the held-out test set. Both RSF and GBSA models demonstrated comparable prognostic performance, with RSF slightly outperforming across most metrics. RSF yielded a mean *C*-index of 0.6082 (SD = 0.0122), compared to 0.6026 (SD = 0.0141) for GBSA. Mean time-dependent AUC values were 0.6174 (RSF) and 0.6119 (GBSA), both exceeding the traditional pathway-based score (AUC range: 0.576–0.613). However, paired *t*-tests revealed no statistically significant differences between models (*P* > .05), suggesting equivalent performance (Figs 5 and 6).

SHAP analysis confirmed the primacy of p53 pathway mutation, TMB, and pathway burden as the top contributors to model predictions in both approaches. In RSF, the mean SHAP values were 32.08 (p53), 15.10 (TMB), and 8.20 (pathway burden). GBSA values followed similar patterns but with generally lower magnitude. Adjusted SHAP comparisons revealed that RSF gave more weight to RTK–RAS and WNT pathways (*P* < .05), while GBSA emphasized TMB (*P* = .0205).

### 3.4 External validation (TCGA cohort)

#### 3.4.1 Patient cohort and clinical characteristics

A total of 501 prostate cancer cases were initially retrieved from The Cancer Genome Atlas (TCGA) database. Patients lacking mutation data were excluded from downstream analyses. The final analytical cohort therefore comprised 466 patients with prostatic adenocarcinoma and available genomic data.

Clinical characteristics were derived directly from the TCGA clinical dataset. The median age at diagnosis was 61 years (interquartile range [IQR]: 56–66). Tumor grade distribution was determined using the primary Gleason grade variable. Based on this classification, 59.4% of tumors were Gleason ≤7, 12.0% were Gleason 8, and 28.6% were Gleason ≥9, indicating a cohort enriched for intermediate-to-high grade disease.

The median OS was 17.1 months (range: 0.03–153.47 months). At the time of data cutoff, 1.7% of patients were recorded as deceased. Collectively, these characteristics reflect a clinically heterogeneous cohort suitable for integrative genomic and pathway-based analyses in prostate cancer.

#### 3.4.2 TMB and gene-level mutation landscape

TMB, defined as the number of distinct mutated genes per patient, was 31 (range: 27–128). Mutation frequency analysis revealed several recurrently altered genes across the cohort, reflecting the genomic heterogeneity characteristic of prostate adenocarcinoma (Table 7).

**Table 7:** Recurrently mutated genes in the TCGA cohort.

Gene	Mutated cases (*n*)	Frequency (%)
*TP53*	52	11.18
*SPOP*	50	10.75
*KMT2D*	28	6.02
*FOXA1*	27	5.81
*KMT2C*	20	4.30
*ATM*	18	3.87
*PTEN*	16	3.44
*KDM6A*	15	3.23
*MUC17*	14	3.01
*APC*	11	2.37
*ZFHX3*	10	2.15
*PIK3CA*	9	1.94
*FLG*	9	1.94
*MXRA5*	8	1.72
*SACS*	8	1.72

#### 3.4.3 Co-occurrence and mutual exclusivity of mutated genes

Pairwise Fisher exact testing identified significant differences in mutation frequencies across these genomic risk groups. The most prominent association involved TP53, which was absent in the low-risk group but present in 29.2% of the intermediate-risk and 50.0% of high-risk tumors, remaining significant after false discovery correction in both comparisons. ATM and PTEN were also significantly enriched in higher-risk tumors. Additional genes significantly enriched in the high-risk group relative to the low-risk group included CTNNB1, APC, CHD4, NCOR1, PIK3CA, SMAD4, CREBBP, EP300, FGFR3, MET, NF1, POLE, BRAF, and KMT2A. No gene remained significantly different between the intermediate- and high-risk groups after multiple-testing correction.

**Figure 7:**
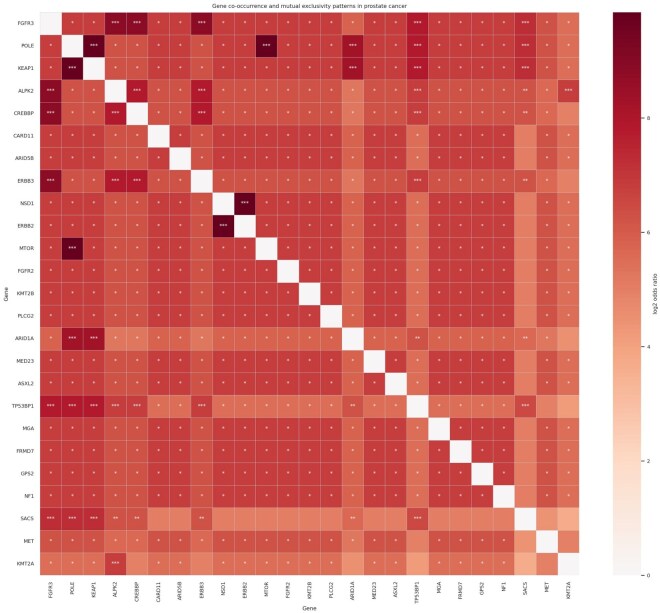
Heatmap displaying pairwise gene co-occurrence and mutual exclusivity based on log2 odds ratios from the TCGA prostate cancer dataset, with asterisks denoting statistically significant gene pairs.

The enrichment heat map demonstrated a consistent increase in recurrent driver alterations across the higher genomic risk groups, with the strongest signal centered on TP53 and secondary contributions from genes involved in DNA repair, chromatin remodeling, and oncogenic signaling pathways. Gene-by-gene concurrence analysis of the top quartile of recurrently altered genes further demonstrated structured patterns of co-occurrence and mutual exclusivity involving TP53, SPOP, KMT2D, FOXA1, ATM, PTEN, APC, and PIK3CA, supporting the presence of biologically distinct genomic programs within the cohort (Fig. 7).

#### 3.4.4 Pathway alteration burden across the cohort

Pathway-level mutational burden was quantified for each patient using the predefined pathway gene sets, and the number of mutated genes within each pathway was recorded. Across the cohort, pathway alterations were unevenly distributed, with a clear predominance of mutations affecting canonical oncogenic signaling cascades.

Alterations involving the TP53 pathway were the most frequent, observed in 67 patients, with a total of 70 mutations identified within this pathway. The RTK–RAS signaling pathway represented the second most frequently altered pathway, with 34 patients harboring at least one mutation (total mutations: 49). Mutations affecting the PI3K pathway were detected in 32 patients (total mutations: 34), while WNT pathway alterations were present in 24 patients (total mutations: 27).

Intermediate mutation frequencies were observed in the NOTCH pathway, which was altered in 18 patients (total mutations: 24), followed by the TGF-β signaling pathway, altered in eight patients (total mutations: 9). Mutations involving cell-cycle regulatory genes were detected in nine patients (total mutations: 11).

In contrast, pathway alterations were relatively rare in several signaling modules. The HIPPO, MYC, and NRF2 pathways each showed mutations in three patients, with total mutation counts of 3, 4, and 4, respectively.

Collectively, these findings indicate that genomic alterations within the cohort are predominantly concentrated in TP53, RTK–RAS, and PI3K signaling networks, whereas disruptions in HIPPO, MYC, and NRF2 pathways occur only sporadically. This distribution highlights the central role of canonical tumor-suppressor and growth-factor signaling pathways in the mutational landscape of the analyzed cohort.

#### 3.4.5 Construction and distribution of the PIRSP-PC

The PIRSP-PC scoring algorithm developed in the MSK-IMPACT cohort was applied without modification to the TCGA dataset to evaluate its genomic generalizability. For each patient, pathway alteration burden, p53 pathway status, and high-risk co-alterations involving PI3K or RTK–RAS signaling were computed using the same annotation framework.

The resulting composite score ranged from 0 to 5, allowing stratification into low-, intermediate-, and high-risk genomic categories according to the predefined thresholds. The distribution of PIRSP-PC scores in the TCGA cohort demonstrated a pattern broadly consistent with that observed in the development cohort, with the majority of tumors clustering in the intermediate and high genomic risk categories.

Importantly, the genomic composition of the high-risk group was characterized by a marked enrichment of TP53 pathway alterations and co-activation of oncogenic signaling pathways including PI3K and RTK–RAS. These findings support the biological reproducibility of the PIRSP-PC framework across independent sequencing datasets.

## 4 Discussion

In this study, we developed and evaluated a pathway-based genomic risk scoring system for prostate adenocarcinoma using a large, clinically annotated cohort from the MSK-IMPACT dataset. By integrating three core dimensions—pathway-level alteration burden, p53 pathway status, and high-risk co-alteration patterns involving the PI3K and RTK–RAS pathways—we constructed a scalable model that provides moderate yet consistent prognostic value for OS.

Our findings support growing evidence that traditional mutation burden metrics such as TMB alone are insufficient for risk stratification in prostate cancer, a disease characterized by relatively low mutational load but substantial biological heterogeneity (Wang *et al.* 2021). Although TMB showed limited discriminative ability in our cohort (AUC ≈ 0.50–0.58), pathway-level functional abstraction enabled extraction of more informative patterns from the same genomic data. These observations suggest that TMB may carry prognostic value primarily when interpreted in combination with additional genomic features.

Among individual predictors, the p53 signaling pathway emerged as the strongest determinant of poor outcome, consistent with prior studies linking TP53 alterations to genomic instability, treatment resistance, and aggressive tumor behavior (Nyquist *et al.* 2020, Ofner *et al.* 2025). Patients with p53 pathway mutations exhibited significantly reduced survival and were overrepresented in the high-risk category. Moreover, concurrent alterations involving p53 and PI3K or RTK–RAS pathways were enriched in high-grade tumors and correlated with increased mutational burden, suggesting cooperative effects that may contribute to aggressive molecular phenotypes (Shorning *et al.* 2020).

By assigning additive weights to these biologically grounded features, PIRSP-PC stratified patients into three risk groups with clearly separated survival curves. High-risk patients (score ≥2) demonstrated substantially poorer outcomes compared with low-risk individuals. Although the model achieved only moderate discrimination (*C*-index ≈ 0.5897), the separation between risk groups was statistically significant and corresponded to a nearly 20-month difference in median survival. These findings indicate that the scoring framework may capture prognostic information beyond commonly used genomic indicators, although its clinical relevance will require validation in independent cohorts.

In multivariable Cox analysis, each one-point increase in risk score was associated with a 31% increase in mortality risk (HR 1.31; 95% CI 1.21–1.42; *P* < .0001). Patients with higher scores showed progressively worse outcomes, suggesting that the score may reflect incremental increases in biological risk. Discrimination and reclassification analyses further supported the added value of the composite score relative to individual predictors. The PIRSP-PC achieved a *C*-index of 0.5897 compared with 0.594 for p53 status alone and 0.571 for TMB, while the Net Reclassification Index at 36 months improved by +0.17 relative to p53 and +0.24 relative to pathway burden.

Machine-learning survival models constructed using the same variables yielded only marginally higher performance (*C*-index ≈ 0.61), indicating that the principal prognostic signal resides largely in p53 status, pathway disruption, and mutational burden. Consistent with this interpretation, SHAP analysis demonstrated strong concordance in feature importance between machine-learning and traditional models. Although ensemble models such as RSFs and gradient boosting offered minor numerical improvements, they did so at the expense of interpretability. In contrast, the PIRSP-PC provides a transparent and easily interpretable framework while achieving comparable predictive performance.

External validation of survival models requires adequate event numbers. In the TCGA cohort, only a small fraction of patients was recorded as deceased at the time of data freeze (∼1.7%), precluding reliable survival modeling. Consequently, TCGA data were used for genomic validation rather than survival analysis, enabling assessment of pathway alteration patterns and distribution of PIRSP-PC risk categories across an independent sequencing platform.

A notable feature of the proposed model is that it relies exclusively on somatic mutation data rather than transcriptomic or epigenomic inputs. This design may facilitate implementation within targeted-sequencing workflows commonly used in clinical oncology, including platforms such as MSK-IMPACT or FoundationOne. The relatively simple structure of the scoring framework—based on pathway alteration burden, p53 status, and biologically motivated co-alteration patterns—may also facilitate evaluation within integrative prognostic models, including future nomogram-based approaches.

Despite these strengths, the discriminatory performance of PIRSP-PC remained modest. Several factors may explain this observation. Prostate cancer survival is influenced by numerous determinants beyond somatic mutation architecture, including treatment exposure, disease burden, clinical stage, androgen receptor signaling, comorbidity, and other non-genomic variables not captured in the present dataset. Additionally, pathway-level abstraction, while improving interpretability, may compress heterogeneous biological events into broader functional categories, potentially reducing prognostic granularity. Furthermore, OS in prostate cancer reflects complex disease trajectories and competing mortality risks, which may limit the predictive resolution of purely genomic models.

This study has several strengths. The analysis leveraged a large and clinically diverse cohort of more than 2000 patients, providing substantial statistical power to detect genomic associations with survival. The pathway-based framework allows biologically interpretable modeling of genomic alterations, in contrast to opaque machine-learning predictors. In addition, the model was evaluated across multiple analytical dimensions—including categorical and continuous risk modeling, time-dependent ROC analysis, and multivariable Cox regression—while machine-learning approaches were used to benchmark performance. All analyses were conducted using reproducible open-source computational environments, enabling transparency and reproducibility.

Nevertheless, several limitations should be acknowledged. The retrospective design introduces potential selection bias and limits control over confounding variables. The MSK-IMPACT panel, while clinically validated, captures only coding mutations within a predefined set of cancer genes, excluding potentially relevant non-coding variants, structural alterations, and epigenetic events. Detailed treatment information was also unavailable, restricting assessment of predictive performance within specific therapeutic contexts. Direct numerical comparisons with commercial transcriptomic classifiers such as Decipher, Prolaris, or Oncotype DX GPS were therefore not feasible because their proprietary inputs were not available in the dataset. Finally, although the model demonstrated internal robustness through bootstrap resampling and train—test validation, its generalizability remains to be confirmed in independent datasets. In addition, the absence of harmonized variables required for formal CAPRA-, NCCN-, or STAR-CAP-based recalibration, together with the lack of systematic germline annotation and dedicated DDR subclassification, limited our ability to determine whether PIRSP-PC adds independent prognostic value beyond established clinical frameworks or captures therapeutically relevant hereditary repair defects.

From a biological perspective, our results emphasize the central role of p53 pathway disruption in shaping prostate cancer prognosis, particularly when combined with alterations in mitogenic signaling pathways such as PI3K and RTK–RAS. These observations are consistent with experimental studies demonstrating that p53 loss promotes lineage plasticity, resistance to androgen-targeted therapy, and neuroendocrine differentiation (Beltran *et al.* 2019). More broadly, the results suggest that pathway-level genomic architecture may capture much of the prognostic signal identified by more complex non-linear survival models.

The novelty of PIRSP-PC lies not in the general concept of genomic risk stratification but in its mutation-based design and translational simplicity. Unlike established transcriptomic classifiers—including Decipher, Prolaris, Oncotype DX GPS, PAM50, and PCS—the model relies solely on somatic mutation data and therefore does not require expression profiling, platform-specific normalization, or proprietary coefficients. Rather than replacing existing classifiers, PIRSP-PC aims to provide a transparent mutation-based alternative compatible with routine targeted-sequencing workflows.

This positioning is particularly relevant when PIRSP-PC is considered alongside established clinicopathologic frameworks such as CAPRA, NCCN risk groups, and STAR-CAP. CAPRA was developed as a pragmatic pretreatment instrument integrating PSA, Gleason score, clinical T stage, age, and biopsy core involvement, and remains valuable because of its simplicity and broad clinical familiarity. Similarly, NCCN risk stratification provides a widely adopted management framework by grouping patients into clinically actionable categories based on PSA, grade, and stage, thereby supporting treatment allocation in localized disease. More recently, STAR-CAP was designed as an AJCC-compliant clinical prognostic staging system for nonmetastatic prostate cancer and demonstrated improved prostate cancer—specific mortality discrimination relative to conventional stage-based approaches in large international cohorts. In this context, PIRSP-PC should not be interpreted as a replacement for these tools. Rather, it addresses a different analytical layer by attempting to capture pathway-level tumor biology from somatic sequencing data, particularly in settings where clinicopathologic groupings alone may not fully reflect underlying molecular heterogeneity (Cooperberg *et al.* 2005, Dess *et al.* 2020, Schaeffer *et al.* 2024).

A key distinction is that CAPRA, NCCN, and STAR-CAP were developed primarily from pretreatment clinicopathologic variables and are therefore highly aligned with treatment selection pathways in localized disease. By contrast, PIRSP-PC was derived from somatic alteration architecture in a large targeted-sequencing cohort with mixed clinical states and an OS endpoint. This difference in design has two implications. First, direct numerical comparison between PIRSP-PC and classic clinical tools should be interpreted cautiously because the models are optimized for partially different use cases, disease contexts, and outcomes. Second, the present results are more appropriately viewed as complementary to clinicopathologic risk assessment than competitive with it. A clinically relevant next step would therefore be formal head-to-head and integrated modeling against CAPRA-, NCCN-, and STAR-CAP-based frameworks in datasets containing harmonized stage, PSA, grade, treatment, and longitudinal outcome data. Such analyses would clarify whether pathway-based genomic abstraction provides incremental prognostic information beyond established clinical stratification rather than merely recapitulating it.

An additional issue that warrants consideration is the relationship between somatic pathway-based scoring and the increasingly important germline/DDR axis in prostate cancer. Pathogenic alterations in DNA damage response and repair genes—particularly BRCA2, but also ATM, CHEK2, PALB2, and related homologous recombination genes—have both prognostic and therapeutic implications. Germline BRCA1/2 carriers, especially BRCA2 carriers, have been shown to present with more aggressive clinicopathologic features, a higher likelihood of nodal or metastatic disease, and inferior cancer-specific outcomes. Moreover, a substantial proportion of DDR abnormalities identified in advanced prostate cancer may be germline in origin, which extends the clinical significance of molecular testing beyond tumor biology alone to familial counseling, cascade testing, and treatment selection.

Within this context, PIRSP-PC captures part of the downstream biological consequence of genomic disruption, but it does not explicitly distinguish somatic from germline events and it was not designed to model DDR-deficient states as a dedicated clinical subgroup. This is an important boundary of interpretation. DDR alterations may contribute to prognosis not only through generalized pathway burden, but also through lineage-specific therapeutic vulnerabilities, including sensitivity to PARP inhibition, platinum-based strategies, and selected immunotherapy contexts. Accordingly, future development of PIRSP-PC could be strengthened by incorporating dedicated annotation of DDR pathway status, BRCA2-enriched subgroups, and germline-versus-somatic origin when available. Such integration may be particularly valuable for testing whether pathway-level risk and DDR-defined therapeutic vulnerability represent overlapping or orthogonal dimensions of prostate cancer biology.

Future studies should focus on prospective validation in multi-institutional cohorts with standardized clinical follow-up. Integrating genomic information with transcriptomic, proteomic, or clinical variables may further refine risk stratification and improve prediction of therapeutic response. Linking risk scores with treatment outcomes—particularly responses to targeted therapies and immunotherapeutic strategies—will also be important for evaluating the potential role of this framework in precision oncology.

## 5 Conclusion

In summary, this study defines and validates PIRSP-PC as a biologically grounded, pathway-centric genomic risk-stratification framework for prostate adenocarcinoma. By integrating cumulative pathway disruption with p53 dysfunction and high-risk PI3K and RTK—RAS co-alterations, PIRSP-PC captures higher-order oncogenic architecture that is not adequately reflected by mutation burden—based metrics or single-gene indicators. The score demonstrates reproducible, time-dependent prognostic discrimination for OS and retains independent prognostic significance after adjustment for established clinicopathologic variables, underscoring its incremental value beyond conventional risk models.

Notably, concordance between PIRSP-PC and non-linear survival machine-learning approaches, both in performance and in the prioritization of dominant biological drivers, supports the stability and biological plausibility of the underlying feature selection. This convergence suggests that transparent, rule-based genomic modeling can achieve prognostic resolution comparable to more complex black-box frameworks while preserving interpretability—a prerequisite for clinical translation.

From a translational perspective, PIRSP-PC leverages routinely generated targeted-sequencing data and provides a scalable platform for genomic risk refinement in prostate cancer. Although external validation and treatment-stratified analyses are warranted, these findings support PIRSP-PC as a candidate tool for biologically informed risk stratification, hypothesis generation, and prospective integration into precision oncology workflows.

## Supplementary Material

vbag093_Supplementary_Data

## Data Availability

All datasets analyzed in this study are publicly accessible from cBioPortal (https://www.cbioportal.org/study/summary?id=prostate_msk_2024 and https://www.cbioportal.org/study/summary?id=prad_tcga_gdc).

## Trial registration

Not applicable, as this study involved retrospective analysis of publicly available, de-identified data without any interventional component.
